# Case Report: Synergistic effects of an *ASXL3* mutation and a 15q11.2 BP1-BP2 microdeletion in a severe neurodevelopmental phenotype

**DOI:** 10.3389/fgene.2025.1674158

**Published:** 2025-12-11

**Authors:** Mingkai Yang, Yanfang Xiao, Chanjuan Chen, Zhou Chu, Guohong Hu

**Affiliations:** 1 Department of Pediatrics, Zhuzhou Clinical College, Jishou University, Zhuzhou, Hunan, China; 2 Department of Pediatrics, Zhuzhou Central Hospital, Zhuzhou, Hunan, China

**Keywords:** dual molecular diagnosis, ASXL3, 15q11.2 BP1-BP2 microdeletion, multilocus pathogenic variation, neurodevelopmental disorder

## Abstract

**Background:**

Bainbridge–Ropers syndrome (BRPS, OMIM #615485) and the 15q11.2 BP1-BP2 microdeletion syndrome (OMIM #615656) are distinct genetic aetiologies of neurodevelopmental disorder Dual diagnosis of both entities in a single patient is extremely rare, and the underlying synergistic pathogenesis remains poorly understood.

**Methods:**

We report a 7-month-old boy presenting with severe global developmental delay, hypotonia, feeding difficulties, microcephaly and recurrent respiratory infections. Whole-exome sequencing (WES) was performed and a protein–protein-interaction (PPI) network was constructed using the STRING database to aid molecular diagnosis. Clinical management and 7-month outcome are described.

**Results:**

WES identified a *de novo* nonsense mutation in *ASXL3* (c.1094C>G, p. Ser365*) and a 1.22-Mb 15q11.2 microdeletion (BP1-BP2) inherited from the asymptomatic father, establishing a dual diagnosis. The PPI network revealed no direct or high-confidence (>0.4) interactions between *ASXL3* and the 15q11.2 BP1-BP2 microdeletion-encoded proteins *CYFIP1*, *NIPA1*, *NIPA2* or *TUBGCP5*, indicating convergence at the pathway rather than the complex level.

**Conclusion:**

The 15q11.2 BP1-BP2 microdeletion acts as a genetic modifier that may amplify the phenotypic expression caused by the core mutation in the ASXL3 gene. Haploinsufficiency of *CYFIP1*, *NIPA1*, *NIPA2*, and *TUBGCP5* increases neurodevelopmental susceptibility, while the *de novo* truncating mutation in *ASXL3* drives severe epigenetic dysregulation. Together, they precipitate the profound phenotype observed here. This case suggests that multilocus pathogenic variation can generate a blended, severe phenotype and underscores the need to consider polygenic burden plus gene–environment interactions in complex NDD. We proposed a “core mutation - gene regulator - environment” synergy hypothesis model, which is of significant guidance value for genetic counseling and personalized clinical management.

## Introduction

Bainbridge–Ropers syndrome (BRPS, OMIM #615485) is a rare autosomal-dominant neurodevelopmental disorder caused by *de novo* heterozygous mutations in *ASXL3*. Core features include profound developmental delay, intellectual disability, hypotonia, feeding difficulties, a characteristic facial gestalt and autistic-like behaviours (Siu Xiao et al., 2022) (Table 1).

**TABLE 1:** Comparison of clinical features between typical Bainbridge-Ropers syndrome, 15q11.2 BP1-BP2 microdeletion syndrome, and the current proband.

Clinical feature	Typical BRPS (ASXL3 mutation)	15q11.2 BP1-BP2 (Risk spectrum)	Current proband (Dual diagnosis)	Interpretation in our case
Global developmental delay (incl. Motor and speech)	∼100%	73% (DD)	Present (severe)	Core feature of both, severity dramatically amplified in proband
Speech delay	100%	67%	Present (severe, no visual tracking)	Core feature of BRPS, severity amplified
Hypotonia	82%	Not a hallmark	Present (profound)	Core and severe feature of BRPS
Feeding difficulties	90%	Not a hallmark	Present (profound, requiring NG tube)	Core and severe feature of BRPS
Characteristic facies	98%	46% (ear/palate anomalies)	Present (prominent forehead, hypertelorism, etc.)	Present in both, risk potentially compounded
Seizures/EEG abnormal	38%	26%	Present (abnormal aEEG)	Core feature of BRPS, severity amplified
Microcephaly	32%	Not a hallmark	Present (Z < −3 SD)	Primarily associated with severe BRPS
Recurrent infections	Not typical	Not typically highlighted	Present (profound, 9 hospitalizations)	A novel severe feature, potentially from synergistic immune-neural mechanism
GERD	82%	Not a hallmark	Present (severe)	Primarily associated with severe BRPS
Behavioral Issues/ASD	75%	27%	Too young to assess	N/A

The prevalence data for typical BRPS, are compiled from references (Balasubramanian et al., 2017; Ling et al., 2024). The prevalence data for the 15q11.2 BP1-BP2 microdeletion syndrome are compiled from references (Cox and Butler, 2015; Butler, 2023). “Not a hallmark” indicates that the feature is not a commonly reported or defining characteristic of the syndrome. GDD, global developmental delay; ID, intellectual disability.

The 15q11.2 BP1-BP2 microdeletion syndrome (OMIM #615656) is a distinct genomic disorder encompassing four highly conserved genes—*CYFIP1*, *NIPA1*, *NIPA2* and *TUBGCP5* (Rafi and Butler, 2020). The deletion exhibits incomplete penetrance and variable expressivity; most carriers are clinically asymptomatic, yet the microdeletion is recognised to confer increased susceptibility to neurodevelopmental conditions such as intellectual disability, schizophrenia and epilepsy (Cox and Butler, 2015) (Table 1).

In 2018, Karaca et al. (2018) cited an individual harbouring both an *ASXL3* mutation and the 15q11.2 BP1-BP2 microdeletion who manifested a more complex phenotype—developmental delay/intellectual disability, epilepsy, microcephaly, diffuse cortical atrophy and gastro-oesophageal reflux—and presented this case as key support for the “multilocus pathogenic variation” hypothesis. However, no systematic clinical report of such a dual diagnosis has been published. Whether the two lesions functionally interact or simply additively combine remains unresolved.

Here we describe a 7-month-old boy in whom whole-exome sequencing (WES) simultaneously identified a *de novo ASXL3* nonsense mutation and the 15q11.2 BP1-BP2 microdeletion. By delineating his severe blended phenotype and reviewing the molecular literature, we explore the potential synergistic interaction between these variants, emphasise the impact of multilocus pathogenic burden and gene–environment interplay in complex neurodevelopmental disorders, and provide practical insights for genetic counselling and clinical management of similarly rare dual diagnoses.

## Case information

**FIGURE 1:**
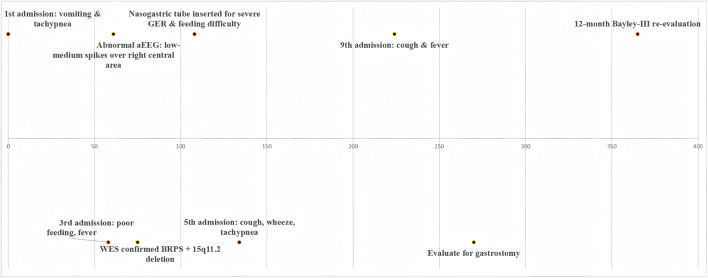
The main timeline of the proband.

The proband, a boy aged 7 months and 3 days, was admitted for the ninth time with a 1-day history of cough and 1 h of fever (Figure 1). From the neonatal period he exhibited progressively worsening feeding difficulties and discoordinated swallowing accompanied by severe gastro-oesophageal reflux, necessitating nasogastric tube feeding. Concomitant tracheomalacia (aryepiglottic and soft-palate collapse) caused airway obstruction and retention of secretions, predisposing him to recurrent severe respiratory infections that had already prompted multiple previous hospitalisations.

On prenatal and family history, G2P2, cesarean section for scar uterus; birth weight 3.4 kg at 39+3 weeks. Parents non-consanguineous, advanced age (father 42 years, mother 38 years). Mother had ovarian cystectomy, hypothyroidism, gestational diabetes and candidal vaginitis; no regular folic acid. Healthy 16-year-old sister; no family history of neurodevelopmental disorders.

**FIGURE 2:**
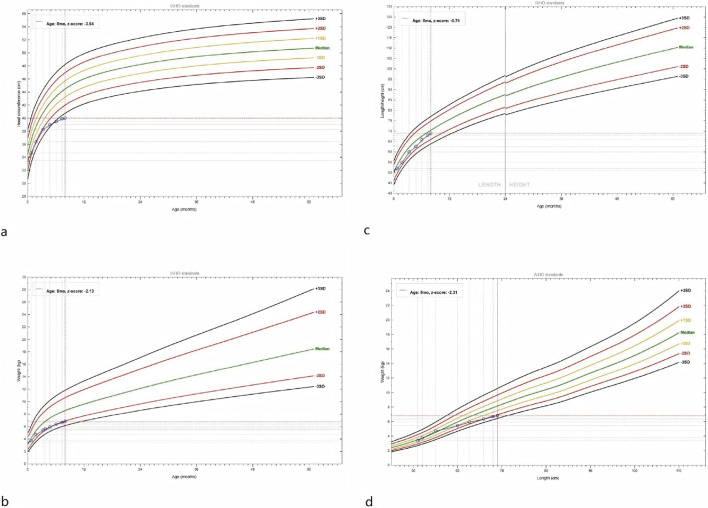
At 7 months the infant exhibited generalized growth failure: **(a)** Head circumference was markedly reduced (Z-score −3.64). **(b)** Weight was moderately low (Z-score −2.13). **(c)** Length bordered the lower limit of normal (Z-score −0.74). **(d)** Weight for length was reduced (Z-score −2.31).

Birth parameters were unremarkable (gestational age 39^+3^ weeks, birth weight 3,400 g), but subsequent growth indices fell substantially below the norm (Figure 2). At 7 months his weight was 6.67 kg (Z = −2.13), length 68 cm (Z = −0.74), and occipito-frontal circumference 39.7 cm (Z = −3.64, < −3 SD), fulfilling the criteria for microcephaly (A Z-score < -3 SD meets the WHO definition of microcephaly, indicating that the occipito-frontal circumference is more than three standard deviations below the age- and sex-specific mean). At 3 months of age, Gesell Developmental Diagnosis showed a Developmental Quotient (DQ) of 70 in adaptive behavior, 63 in gross motor, 63 in fine motor, 55 in language, and 63 in personal-social skills.

On physical examination, dysmorphic face (broad forehead, hypertelorism, high-arched palate, low-set ears, anteverted nares), mild retractions, inspiratory stridor and bilateral crackles. Neurology: generalized hypotonia, limb strength 4/5, absent knee jerks, no head control, no rolling or eye contact.

Ancillary investigations revealed moderate hydronephrosis and mild transaminase elevation in the neonatal period; perinatal CMV-IgM was positive. At 1 month 30 days, amplitude-integrated EEG (aEEG) was abnormal, showing sporadic low-to medium-amplitude spikes and sharp-and-slow waves over the right central region during wakefulness. Non-contrast brain MRI and diffusion-weighted imaging (DWI) disclosed no structural or restricted-diffusion lesions. No clinical seizures had been observed by 7 months of age.

## Genetic analyses

Methods: With parental informed consent, peripheral blood samples (2 mL) were collected from the proband and both parents for trio-based whole-exome sequencing (WES). Genomic DNA extracted from peripheral blood was fragmented and used for library preparation. Target enrichment of exonic regions and flanking splice sites was performed using the Roche KAPA HyperExome kit. Sequencing was carried out on the MGISEQ-2000 or DNBSEQ-T7 platform. Quality control metrics: mean target depth ≥200×, with >98.5% of target bases covered at ≥20×. Reads were aligned to the UCSC hg19 reference genome using BWA. Duplicate reads were removed. SNVs and indels were called using GATK after base quality recalibration. CNVs were detected at the exon level using ExomeDepth. Variant annotation and filtering were performed based on the patient’s clinical phenotype, population databases, and predictive tools. Variant pathogenicity was classified according to the ACMG/AMP guidelines.

**FIGURE 3:**
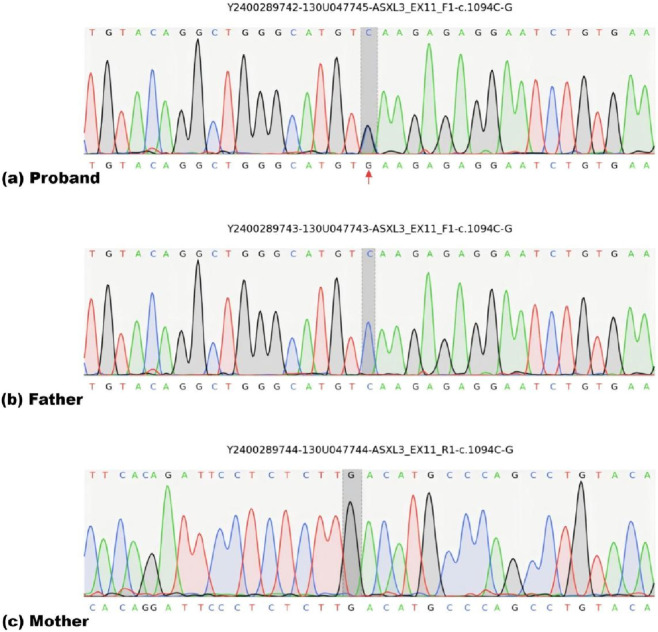
Sanger sequencing of the *ASXL3* variant in the patient and his parents. **(a)** The *ASXL3* variant (NM_030632.1: c.1094C>G, p. Ser365*) at chr18:31 318 462 is detected in the patient, The variant sites are marked with arrows in the figure. **(b)** The variant is absent in the father. **(c)** The variant is absent in the monther (wild-type sequence on the reverse-complementary strand; the actual genomic sequence is identical to the father).

1. A *de novo* nonsense mutation in *ASXL3*: A heterozygous nonsense mutation in *ASXL3* (chr18:31,318,462; c.1094C>G, p. Ser365*) was identified and confirmed by Sanger sequencing; the variant was absent in both parents (Figure 3), consistent with a *de novo* origin. With a population frequency of 0 and fulfilling ACMG criteria (PVS1 + PS2 + PM2), it is classified as pathogenic and fully explains the clinical diagnosis of Bainbridge–Ropers syndrome.
2. 15q11.2 microdeletion: an ∼1.22-Mb loss at chr15:22,382 472–23 604,356 (BP1-BP2) inherited from the asymptomatic father. The segment is a ClinGen-level 3 dosage-sensitive region (HI:3) encompassing *TUBGCP5*, *CYFIP1*, *NIPA1* and *NIPA2.* This deletion is a recognised susceptibility locus for neurodevelopmental impairment (intellectual disability, language delay, ASD) and is classified as pathogenic on in the context of this patient’s dual diagnosis the basis of increased risk and phenotypic fit.
3. Additional variants: *LPIN1* (NM_145693.2:c.2162dupA) and *TMEM63A* (NM_014698.2:c.1571G>C) were rated VUS. *LPIN1* is predicted likely pathogenic (PVS1 + PM2) and *TMEM63A* VUS (PM2 + PP3); however, both are heterozygous, incompatible with the patient’s phenotype, and their associated disorders (acute myoglobinuria, leukodystrophy) are not represented. They are therefore considered secondary findings.

## Protein–protein interaction analysis

**FIGURE 4:**
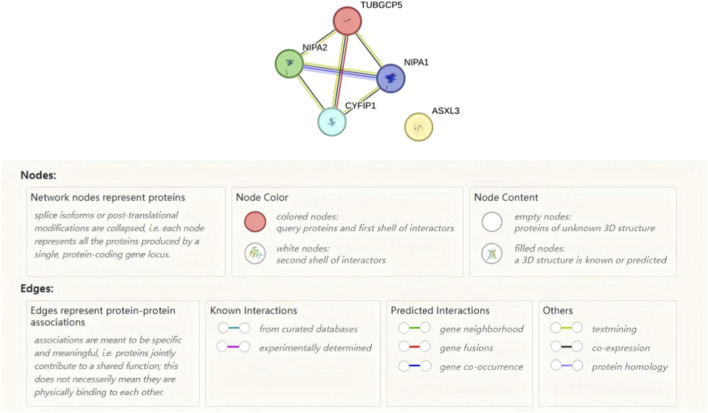
Analysis with the STRING database (https://string-db.org) confirmed the absence of any experimentally validated or high-confidence predicted protein–protein interactions (PPIs) among ASXL3 and the 15q11.2 BP1-BP2-encoded proteins TUBGCP5, CYFIP1, and NIPA1/NIPA2.

*ASXL3*, *TUBGCP5*, *CYFIP1*, *NIPA1* and *NIPA2* were queried in the STRING database (confidence threshold >0.4). As illustrated in Figure 4, no direct or high-confidence interactions were detected between the nuclear epigenetic regulator ASXL3 and the predominantly cytoplasmic/membrane proteins encoded within the 15q11.2 region, indicating that synergism is mediated at the pathway level rather than through shared physical complexes.

## Long-term management and prognosis

A personalised, multidisciplinary care plan was established for the patient’s severe neurodevelopmental delay, feeding difficulties, EEG abnormalities and recurrent infections; the regimen will be adjusted as the child grows.

### Neurodevelopmental delay

Intensive, individualised physiotherapy, occupational therapy and speech-language intervention are ongoing. At 7 months the infant still cannot hold his head steady, roll over or fixate, indicating minimal response to date. Outcome remains guarded; formal scales (Gesell/Bayley-III) will be repeated every 6–12 months to recalibrate goals.

### Feeding difficulty and gastro-oesophageal reflux

A multidisciplinary team (paediatric gastroenterology, nutrition, rehabilitation) supervises swallowing rehabilitation and nutritional optimisation; nasogastric feeding is still required. If aspiration persists despite thickened feeds and proton-pump inhibition, we will reassess for gastrostomy ± fundoplication to reduce recurrent pneumonias.

### Recurrent infections

When planning the next hospitalization, we will complete the tests for quantitative immunoglobulins, lymphocyte subgroups, and vaccine-specific antibody titers after obtaining the consent of the family members. If deficits are found, prophylactic antibiotics or immunomodulation (IVIG/SCIG) will be considered, together with strict adherence to the routine and additional (influenza, pneumococcal, COVID-19, RSV monoclonal) vaccination schedule.

### EEG abnormality

Current tracing shows rare right-central spikes below the anticonvulsant threshold; therefore no medication is started. Repeat EEG is scheduled every 6 months and parents have been trained to recognise possible seizures.

### Future directions

The family will be followed in our neurogenetics clinic with annual multidisciplinary review. We will monitor emerging literature on BRPS and 15q11.2 BP1-BP2-related therapies and consider enrolment in relevant trials to explore potential therapeutic options.

## Discussion

We report an infant with profound neurodevelopmental impairment in whom trio-based WES simultaneously uncovered a pathogenic, *de novo ASXL3* nonsense mutation and a hemizygous 15q11.2 BP1-BP2 microdeletion. This rare “dual diagnosis” is not a simple additive combination of two independent entities; rather, it might be suggesting how a fully penetrant “core” variant and an incompletely penetrant “modifier” allele interact to generate a severe blended phenotype (Posey et al., 2017). The findings may furnish in-vivo evidence for the multilocus pathogenic variation hypothesis, implying that cumulative genetic burden can yield a phenotype markedly more severe than that produced by either lesion alone (Karaca et al., 2018).

The disease caused by *ASXL3* mutation is called Bainbridge–Ropers syndrome—also known as *ASXL3*-related disorder—it is an autosomal dominant genetic disorder caused by a mutation in the *ASXL3* gene at the 18q12.1 region. BRPS is characterised by speech delay, intellectual disability, behavioural anomalies, feeding difficulty, hypotonia, dysmorphism, strabismus and epilepsy (Wang et al., 2022); the patient’s severe feeding problems, hypotonia and global developmental failure map precisely onto this phenotype, establishing *ASXL3* loss-of-function as the primary pathogenic driver.

Superimposed on core *ASXL3* mutation is a 1.22-Mb microdeletion at 15q11.2 BP1-BP2 inherited from the asymptomatic father. This copy-number variant could impair a dosage-sensitive interval (HI:3) that defines Burnside-Butler syndrome and is associated with developmental/language delay, ear/palate malformations, memory and literacy deficits (VIQ ≤75), abnormal neuro-imaging, epilepsy, ASD, ADHD, psychosis and motor delay (Butler, 2023). As Cox and Butler (2015) emphasised, this deletion constitutes a low-penetrance susceptibility locus with incomplete penetrance and variable expressivity: 51% of clinically affected carriers inherit the copy-number variant from a phenotypically normal parent, indicating that most individuals possess genetic or environmental buffering capacity that compensates for haplo-insufficiency. However, when additional factors—such as polygenic variants that reduce buffering capacity or adverse environmental exposures—are present, both penetrance and expressivity may shift accordingly (Kingdom and Wright, 2022).

Genotype-phenotype relationships are not simple one-to-one correspondences but are driven by a complex interplay of layered and interdependent gene-environment interactions (Eberhart et al., 2017). Although their individual contributions cannot be quantified in a single case, it is reasonable to postulate that environmental factors—including advanced parental age, maternal metabolic conditions such as gestational diabetes and hypothyroidism, and a potentially adverse intrauterine inflammatory milieu—may also have exacerbated the phenotypic severity.

Thus, we propose a hypothetical “core-mutation–genetic-modifier–environment” synergistic model: the *de novo ASXL3* truncation serves as the “core hit,” the 15q11.2 BP1-BP2 microdeletion may act as a “genetic modifier” that potentially amplifies the phenotype, and a sub-optimal intrauterine environment constitutes the “third strike.” This synergistic combination may contribute to epigenetic dysregulation based on the known function of *ASXL3*, consistent with gene-environment interaction paradigms (Beames and Lipinski, 2020). The clinical validity of this model is supported by persistent developmental milestone delays at 2 months of age and prominent developmental regression—such as the inability to maintain head control, roll over, or establish eye contact by 7 months. This finding may suggest that neurological function continues to decline progressively despite ongoing rehabilitation interventions and long-term management.

**FIGURE 5:**
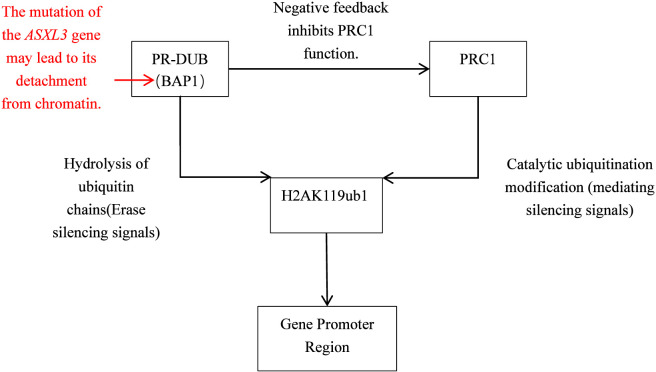
Schematic of how truncating mutations in *ASXL3* disrupts gene expression.

*ASXL3* is highly expressed in human white matter, insula, cingulate gyrus and amygdala (Wu and Cong, 2021). The p. Ser365* nonsense allele creates a truncated protein that can no longer scaffold BAP1 to chromatin (Lichtig et al., 2020). BAP1 is the catalytic sub-unit of the PR-DUB complex that removes ubiquitin from H2AK119; its displacement therefore is predicted to leave H2AK119ub1 enriched at bivalent promoters (Figure 5). Persistent H2AK119ub1 recruits PRC1-mediated silencers, condenses chromatin and inappropriately locks neurogenesis-, differentiation- and synaptic-plasticity-related genes into transcriptional repression (Zhang et al., 2025). Based on the preclinical studies on the loss of *ASXL3* function, it can be inferred that the global genomic aberration patterns of these loci may lead to the early-onset and global developmental failure characteristics of Bainbridge-Ropers syndrome.

The 1.22 Mb 15q11.2 BP1-BP2 microdeletion removes four highly conserved, dosage-sensitive genes whose products converge on neurodevelopmental “execution” pathways.

*CYFIP1* binds FMRP and eIF4E to cap-dependent mRNAs, transiently repressing their translation; it also transmits RAC1-GTP signals to the WAVE-regulatory complex, driving Arp2/3-mediated actin polymerisation. Haplo-insufficiency reduces dendritic-spine density, shortens spine necks and destabilises activity-dependent structural plasticity (Ne et al., 2020; Yan et al., 2016).

*NIPA1*/*NIPA2* integral membrane proteins mediate Mg^2+^ influx at synapses and in the distal nephron. Loss-of-function alleles cause ER retention of the transporter, trigger unfolded-protein-response-mediated apoptosis, and downregulate BMP signalling necessary for axonal maintenance (Fang et al., 2023; Zhang et al., 2025). Reduced extracellular Mg^2+^ further disinhibits NMDA receptors, heightening excitotoxic vulnerability.

*TUBGCP5* nucleates γ-tubulin ring complexes on centrosomes and is essential for symmetric division of neural progenitors. Haplo-insufficiency prolongs G2/M transition, decreases the neuronal precursor pool and impairs radial-glial scaffold orientation, compounding the neurogenesis defect already imposed by *ASXL3*-mediated epigenetic repression (Maver et al., 2019; Rafi and Butler, 2020).

Although PPI analysis revealed no direct physical interaction, the *ASXL3* truncating mutation and the 15q11.2 BP1-BP2 microdeletion may exert their effects through functionally convergent pathways in neurodevelopment. We propose that their synergistic action on convergent neurodevelopmental pathways may potentially underlie the patient’s severe phenotype based on the known functions of the involved genes.

The novel truncating mutation of *ASXL3* may contribute to extensive transcriptional dysregulation by affecting the expression of key genes involved in neural development, neurogenesis, differentiation and synaptic function; the 15q11.2 BP1-BP2 microdeletion interferes with cytoskeletal dynamics, local protein translation and ionic homeostasis, and disrupts the critical “executive system”. This dual insult—epigenetic mis-regulation upstream and failure of cytoskeletal and homeostatic processes downstream—could try to explain the global neurological collapse.

The proband’s congenital CMV infection, together with nine hospital admissions for acute respiratory infections between birth and 7 months—each characterized by poor response and prolonged course—constitutes one of the classic warning signs of primary immunodeficiency (PID). This pattern strongly suggests a possible underlying, inborn defect in immune defence that prevents effective clearance of common respiratory pathogens. Likely a major contributing factor of the patient’s recurrent infections is the severe neurodevelopmental impairment—hypotonia and discoordinated swallowing—that produces feeding difficulties and persistent gastro-esophageal reflux, thereby greatly increasing the risk of aspiration pneumonia. However, a multi-factorial overlay is likely: because actin-cytoskeletal remodelling underlies chemotaxis, phagocytosis and migration in neutrophils and macrophages, *CYFIP1* haplo-insufficiency may compromise these basic immune-cell functions by disturbing cytoskeletal dynamics (Rafi and Butler, 2020; Ronzier et al., 2022). We hypothesize that, given the clinical correlation between these variants and the phenotypic features, secondary aspiration (possibly related to *ASXL3* mutations) and primary immune dysregulation (mediated by the 15q11.2 BP1-BP2 microdeletion) are pathogenically linked. Based on this hypothesis, we propose that their combined effect may account for the extremely severe respiratory infection phenotype observed in this infant. Functional studies (e.g., neutrophil chemotaxis assays) will be required to confirm this assumption in the future.

Although the “core-mutation–genetic-modifier–environment” model provides a compelling framework for the proband’s severe phenotype, several limitations must be acknowledged. First, the proposed synergistic pathogenesis, while consistent with the clinical severity, remains a mechanistic hypothesis. The precise molecular crosstalk among the disrupted pathways—epigenetic regulation, cytoskeletal dynamics, and ion homeostasis—is inferred from established literature rather than demonstrated in this patient.

Second, direct experimental evidence is lacking. Most notably, the assertion of ASXL3-mediated epigenetic dysregulation is based on protein function; no genome-wide profiling (e.g., ChIP-seq for H2AK119ub1) was performed to confirm widespread chromatin perturbation.

Third, with only a single reported case, we cannot determine whether this combination represents a recurrent pathogenic entity or a rare coincidence. The incomplete assessment of gene-environment interactions also precludes definitive conclusions about the contribution of adverse prenatal factors.

Therefore, our model should be viewed as hypothesis-generating. Future work should include functional assays in patient-derived neurons and expanded cohort screening to statistically validate this synergy and establish its clinical generalizability.

## Conclusion

We describe a single infant with a dual molecular diagnosis of Bainbridge–Ropers syndrome and the 15q11.2 BP1-BP2 microdeletion whose severe, early-onset neurodevelopmental failure exceeds the expected spectrum of either lesion alone. Our hypothetical “core-mutation–genetic-modifier–environment” model—anchored on a *de novo* ASXL3 truncating allele, a paternally inherited 15q11.2 BP1-BP2 microdeletion, and adverse prenatal factors—provides a testable framework for this exaggerated phenotype, but remains unsupported by direct functional or epigenomic data. With only one patient, we cannot determine whether the observed synergy represents a recurrent pathogenic entity or a rare stochastic coincidence. Large-scale cohorts, patient-derived neuronal models, and murine knock-in/knock-out systems will be required to validate the proposed molecular crosstalk, quantify the contribution of each “hit,” and establish whether this combination constitutes a clinically actionable syndrome rather than an anecdotal coexistence.

## Data Availability

The datasets presented in this article are not readily available because of ethical and privacy restrictions. Requests to access the data that support the findings of this study should be directed to the corresponding author.
