# Correction: Case Report: Synergistic effects of an *ASXL3* mutation and a 15q11.2 BP1-BP2 microdeletion in a severe neurodevelopmental phenotype

**DOI:** 10.3389/fgene.2025.1769021

**Published:** 2026-01-09

**Authors:** Mingkai Yang, Yanfang Xiao, Chanjuan Chen, Zhou Chu, Guohong Hu

**Affiliations:** 1 Department of Pediatrics, Zhuzhou Clinical College, Jishou University, Zhuzhou, Hunan, China; 2 Department of Pediatrics, Zhuzhou Central Hospital, Zhuzhou, Hunan, China

**Keywords:** dual molecular diagnosis, ASXL3, 15q11.2 BP1-BP2 microdeletion, multilocus pathogenic variation, neurodevelopmental disorder

Affiliation “Department of Pediatrics, Zhuzhou Central Hospital, Zhuzhou, Hunan, China” was omitted for authors Yanfang Xiao, Chanjuan Chen and Zhou Chu. This affiliation has now been added for authors Yanfang Xiao, Chanjuan Chen and Zhou Chu.

Authors Yanfang Xiao, Chanjuan Chen and Zhou Chu were erroneously assigned to affiliation “Department of Pediatrics, Zhuzhou Clinical College, Jishou University, Zhuzhou, Hunan, China”. This affiliation has now been removed for authors Yanfang Xiao, Chanjuan Chen and Zhou Chu.

The original article has been updated.

